# Risk Factors and Mortality Rates Associated With Invasive Group B *Streptococcus* Infections Among Patients in the US Veterans Health Administration

**DOI:** 10.1001/jamanetworkopen.2019.18324

**Published:** 2019-12-27

**Authors:** Robin L. P. Jump, Brigid M. Wilson, Daniel Baechle, Janet M. Briggs, Richard E. Banks, Sunah Song, Taissa Zappernick, Federico Perez

**Affiliations:** 1 Geriatric Research Education and Clinical Center, VA Northeast Ohio Healthcare System, Cleveland; 2 Specialty Care Center of Innovation, VA Northeast Ohio Healthcare System, Cleveland; 3 Division of Infectious Diseases and HIV Medicine, Department of Medicine, Case Western Reserve University School of Medicine, Cleveland, Ohio; 4 Department of Population and Quantitative Health Sciences, Case Western Reserve University School of Medicine, Cleveland, Ohio; 5 Cleveland Institute for Computational Biology, Case Western Reserve University School of Medicine, Cleveland, Ohio

## Abstract

**Importance:**

The incidence of invasive infections caused by group B *Streptococcus* (GBS) continues to increase in the United States. Although diabetes is a key risk factor for invasive GBS, the influence of long-term glycemic control is not well characterized; other risk factors and mortality rates associated with specific types of invasive GBS infections are unknown.

**Objective:**

To investigate risk factors and mortality rates associated with specific invasive GBS infectious syndromes.

**Design, Setting, and Participants:**

This cohort study used US Veterans Health Administration data to assess active users of the Veterans Affairs health care system between January 1, 2008, and December 31, 2017. Data analysis was conducted from April 2018 to August 2019.

**Exposures:**

Invasive GBS infections.

**Main Outcomes and Measures:**

The specific types of infectious syndromes and comorbid conditions among patients with an invasive GBS infection were evaluated.

**Results:**

Between 2008 and 2017, 5175 patients in the Veterans Affairs health care system experienced 5497 invasive GBS infections (5027 [97.1%] men, 3737 [72.2%] white, and 4545 [87.8%] non-Latino); all-cause 30-day mortality was 8.7% (451 of 5175). The most frequently observed infections were osteomyelitis (1171 [21.3%]), bacteremia without focus (1009 [18.4%]), skin or soft-tissue infections (919 [16.7%]), and pneumonia or empyema (694 [12.6%]). All-cause 30-day mortality following the index infection for each patient was highest among patients with peritonitis (38 of 138 [27.5%]) and pneumonia or empyema (116 of 664 [17.5%]) and lowest among those with osteomyelitis (15 of 1075 [1.4%]) or joint infection (17 of 501 [3.4%]). The most common comorbid conditions among patients with invasive GBS infections were diabetes (3364 [65.0%]), obesity (2669 [51.6%]), and chronic heart conditions (1633 [31.6%]). From 2008 to 2017, the incidence of invasive GBS infections increased from 9.23 to 11.67 cases per 100 000 person-years (*P* = .049). Stratification by body mass index showed the highest incidence of infections among patients at the extremes of body mass index (body mass index <18.5, 25.1 cases per 100 000 person-years; body mass index ≥40, 31.0 cases per 100 000 person-years). The incidence among patients with diabetes and poor long-term glycemic control (ie, hemoglobin A_1c_ ≥9.5%) was 4-fold greater than among patients with diabetes and good glycemic control (ie, hemoglobin A_1c_ <7.5%) (78.3 cases vs 19.0 cases per 100 000 person-years; unadjusted incident rate ratio, 4.1; 95% CI, 3.7-4.4; *P* < .001).

**Conclusions and Relevance:**

In this cohort study, comorbid conditions and 30-day mortality varied among types of invasive GBS infections. Obesity and poor long-term glycemic control were associated with invasive GBS infections. Efforts to reverse the trend of an increased incidence of invasive GBS infections should continue to support reducing obesity and focus on improving glycemic control.

## Key Points

**Question:**

What are the risk factors and mortality rates associated with different types of invasive group B *Streptococcus *(GBS) infections?

**Findings:**

In this cohort study of 5175 veterans with 5497 cases of invasive GBS infections, all-cause 30-day mortality was highest among patients with peritonitis (28%) and pneumonia or empyema (17%) and lowest among those with osteomyelitis (1%) or joint infection (3%). The incidence of invasive GBS infections among patients with poorly controlled diabetes was 4-fold higher than among patients with well-controlled diabetes.

**Meaning:**

Poor long-term glycemic control was associated with increased risk of invasive GBS infections.

## Teaser

This cohort study investigates risk factors and mortality rates associated with specific invasive group B *Streptococcus* infectious syndromes among patients of the Veterans Affairs health care system.

## Introduction

The incidence of invasive group B *Streptococcus* (GBS) infections among nonpregnant adults in the United States tripled between 1990 and 2016.^1,2^ In the last 3 decades, analyses of population-based data from the US Centers for Disease Control and Prevention Active Bacterial Core surveillance (ABCs) program has consistently identified older age and diabetes mellitus as risk factors for invasive GBS infections, with more recent data indicating an association with obesity as well.^1,2,3,4^ These findings raise additional questions about risk factors and mortality rates associated with specific types of GBS infections. Furthermore, the association of long-term glycemic control among individuals with diabetes with the risk of developing an invasive GBS infection has not been characterized. We postulated that underlying comorbid conditions and outcomes varied according to the type of invasive GBS infection and that the risk of invasive GBS infections was higher among individuals with poor long-term glycemic control and those with extreme obesity. To explore these questions, we used microbiological, clinical, and administrative databases from the US Veterans Health Administration (VHA) to investigate risk factors and mortality associated with different types of invasive GBS infectious syndromes among users of the Veterans Affairs (VA) health care system.

## Methods

### Study Design and Data Sources

The institutional review board at the VA Northeast Ohio Healthcare System approved the study protocol and granted a waiver of informed consent because the research was no more than minimal risk and the waiver would not adversely affect the participants’ rights and welfare. This study followed the Strengthening the Reporting of Observational Studies in Epidemiology (STROBE) reporting guideline.

We conducted a cohort study of patients treated at the VA health care system from January 1, 2008, through December 31, 2017, using the VA Informatics and Computing Infrastructure to access the VHA Corporate Data Warehouse (CDW). Data were extracted from the CDW on April 20, 2018. Cultures growing GBS and the anatomic site of those cultures were identified by searching microbiology tables in the CDW. The following data were also obtained from the CDW: patient demographic characteristics, including height and weight, laboratory values, and, using codes from the *International Classification of Diseases, Ninth Revision (ICD*-*9)* and *ICD-10*, infectious diagnoses and comorbid conditions. Data indicating date of death and Medicare enrollment among VA health care users were extracted from the from the VHA Vital Status File on June 25, 2019.

### Case Ascertainment and Clinical Characteristics

Inclusion criteria were VA health care users with at least 1 case of invasive GBS infection, defined as a culture from a normally sterile site that grew GBS (ie, blood, bone, or cerebrospinal, synovial, pleural, and peritoneal fluids).^5^ Exclusion criteria were patients who were not active users of the VA health care system in the year of the positive culture. Active use was defined as a hospital admission or an outpatient primary or specialty care clinic visit in that year. Among patients with multiple cultures consistent with an invasive GBS infection, a culture was considered a separate case if it occurred at least 30 days after the incident case. Separate cases that occurred in the same patient were considered recurrences.

Only incident cases were included when analyzing age, sex, self-reported race/ethnicity, comorbid conditions, all-cause mortality at 30 days and 1 year, the number of recurrent cases, Charlson Comorbidity Index (CCI) score, body mass index (BMI, calculated as weight in kilograms divided by height in meters squared), percentage of glycated hemoglobin or hemoglobin A_1c_ (HbA_1c_), and type of infectious syndrome. We determined the CCI score using *ICD* codes.^6^ To calculate BMI, we used the first height and weight measurements within the same calendar year as the infection; if these were not available, we used the first BMI from a previous calendar year, using a last-observed, carry-forward logic for missing values. For HbA_1c_ percentage among patients with an *ICD* code for diabetes, we similarly used the first value within the same calendar year as the infection; if the HbA_1c_ percentage was not present during the year, it was deemed a missing value.^4^

To determine the types of invasive GBS infection, we considered the culture site in conjunction with the *ICD* codes associated with each case. Culture sites informed a preliminary classification of cases according to the following hierarchy: cerebrospinal fluid, heart-related, pleural fluid, synovial fluid, bursal fluid, bone, intra-abdominal fluid, and blood. We used *ICD* codes associated with each case to confirm the specific invasive GBS infection syndromes (eTable 1 in the Supplement). We considered outpatient *ICD* codes entered 7 days before to 30 days after a GBS culture to be associated with the given culture. Similarly, we considered inpatient *ICD* codes entered for an admission that included a GBS culture or began within 30 days of a GBS culture to be associated with that culture. We also assessed if the cases were polymicrobial, defined as isolation of bacteria different from GBS in the same sample that grew GBS. Specifically, we recorded the presence of *Staphylococcus aureus, Pseudomonas aeruginosa*, or other bacteria.

Cases of invasive GBS infection in which the anatomical culture site was consistent with the clinical syndrome were considered concordant (eg, cultures from bone or peritoneal fluid were concordant with an *ICD* code for osteomyelitis or intra-abdominal infection, respectively). For cases in which only blood cultures were positive for GBS, we searched *ICD* codes to identify associated infectious syndromes. If the cultures and *ICD* codes did not indicate an infectious syndrome other than bacteremia, the case was designated bacteremia without focus. We classified 2 types of invasive GBS infection cases as discordant: (1) cases identified by a GBS culture but without any *ICD* code indicating an infectious syndrome and (2) cases in which the site of the GBS culture was not consistent with the infectious syndrome indicated by an associated *ICD* code (eg, a patient with a bone culture growing GBS and an *ICD* code for an unrelated infection, such as pneumonia).

### Statistical Analysis

To determine rates of invasive GBS infection among VA patients, we considered all cases (ie, concordant, discordant, incident, and recurrent) and calculated person-years at risk from the cohort of active VA health care users. For each calendar year, each patient who received inpatient or outpatient clinical care in the VA contributed 1 person-year at risk if they were alive at the end of the year; if they died in the given year, they contributed the fraction of the year during which they were alive. The determination of active users for the denominator was made for each year of the study. Overall and age-stratified incidence rates with 95% CIs were calculated using Poisson methods. We performed similar calculations including only VA health care users who were not enrolled in Medicare and compared them with estimates of incidence for different age strata from ABCs data.^5^ Additionally, incidence rates were calculated and compared across strata of BMI including less than 18.5 (ie, underweight) and 40 and greater (ie, extreme obesity). Similarly, incidence rates were calculated for patients without diabetes and for patients with diabetes with different levels of long-term glycemic control according to HbA_1c_ percentage, as follows: less than 7.5% (ie, well controlled), 7.5% to 9.4%, and 9.5% or higher (ie, poorly controlled). We used Poisson regression models to estimate the incidence rate ratios of invasive GBS infection in different age and BMI strata as well as among patients with diabetes and, among patients with diabetes, in different long-term glycemic control strata. Univariate and multivariable models were estimated using the stratum of age, BMI, or glycemic control with the lowest crude incident rate as the reference level. Crude incidence rates across combinations of age, BMI, and HbA_1c_ percentage groups were graphically displayed. Kaplan-Meier survival curves for the year following incident infection were estimated by type of invasive GBS infection; the survival curves of the most common syndromes were compared using a log-rank test. The Mann-Kendall test was used to assess trends for incidence rates over time, with a 2-sided *P* < .05 considered statistically significant. All statistical analyses were performed using R version 3.5.1 (R Project for Statistical Computing) including functions from the epitools, trend, *ICD*, and survival packages.

## Results

### Clinical Characteristics

Between 2008 and 2017, GBS caused 5497 invasive infections among 5175 VA health care users. Most patients were male (5027 [97.1%]), white individuals (3737 [72.2%]), and non-Latino individuals (4545 [87.8%]) (Table 1). The most common invasive GBS infection was osteomyelitis (1171 [21.3%]), followed by bacteremia without focus (1009 [18.4%]), skin or soft-tissue infections (919 [16.7%]), and pneumonia or empyema (694 [12.6%]), which together accounted for nearly 70% of incident cases of invasive GBS infection. An additional 418 patients (7.6%) had a culture indicating an invasive GBS infection without a concordant *ICD* code. Compared with patients with a concordant index case, discordant cases had significantly higher rates of mortality (73 [17.5%] vs 378 [7.9%]; *P* < .001) and malignant neoplasm (114 [27.3%] vs 1034 [21.7%]; *P* = .005) and lower rates of diabetes (215 [51.4%] vs 3149 [66.1%]; *P* < .001) and obesity (171 [40.9%] vs 2498 [52.4%]; *P* < .001).

**Table 1.** Characteristics of Patients in the Veterans Health Administration With Invasive Group B *Streptococcus* Infections, by Age

Characteristic	No. (%)						
	All (N = 5175)	Aged 18-34 y (n = 41)	Aged 35-49 y (n = 249)	Aged 50-64 y (n = 2125)	Aged 65-74 y (n = 1542)	Aged 75-84 y (n = 780)	Aged ≥85 y (n = 438)
Male sex	5027 (97.1)	35 (85.4)	228 (91.6)	2056 (96.8)	1512 (98.1)	774 (99.2)	422 (96.3)
Age, mean (SD), y	66.5 (11.7)	30.3 (3.3)	45.2 (3.3)	58.9 (3.9)	68.7 (2.8)	79.5 (2.9)	88.8 (3.2)
Race							
White	3737 (72.2)	23 (56.1)	159 (63.9)	1440 (67.8)	1176 (76.3)	603 (77.3)	336 (76.7)
Black	1022 (19.7)	14 (34.1)	64 (25.7)	495 (23.3)	253 (16.4)	127 (16.3)	69 (15.8)
Other^a^	416 (8.0)	4 (9.6)	26 (10.4)	190 (8.9)	113 (7.3)	50 (6.4)	33 (7.5)
Ethnicity							
Non-Latino	4545 (87.8)	34 (82.9)	215 (86.3)	1864 (87.7)	1390 (90.1)	672 (86.2)	370 (84.5)
Latino	418 (8.1)	6 (14.6)	28 (11.2)	165 (7.8)	101 (6.5)	71 (9.1)	47 (10.71)
Other^b^	212 (4.1)	1 (2.4)	6 (2.4)	96 (4.5)	51 (3.3)	37 (4.7)	21 (4.8)
Charlson Comorbidity Index score, mean (SD)	3.8 (2.6)	1.7 (1.9)	2.6 (2.1)	3.5 (2.5)	4.1 (2.6)	4.3 (2.7)	4 (2.6)
BMI ≥30	2669 (51.6)	10 (24.4)	132 (53.0)	1174 (55.2)	912 (59.1)	338 (43.3)	103 (23.5)
Diabetes with hemoglobin A_1c_ ≥7.5%	1684 (32.5)	6 (14.6)	102 (41.0)	854 (40.2)	506 (32.8)	168 (21.5)	48 (11.0)
All-cause 30-d mortality	451 (8.7)	2 (4.9)	6 (2.4)	140 (6.6)	134 (8.7)	95 (12.2)	74 (16.9)
Syndrome^c^							
Any invasive infections	5497 (100)	42 (100)	280 (100)	2279 (100)	1625 (100)	819 (100)	452 (100)
Osteomyelitis	1171 (21.3)	9 (21.4)	95 (33.9)	589 (25.8)	346 (21.3)	108 (13.2)	24 (5.3)
Bacteremia without focus	1009 (18.4)	13 (31.0)	25 (8.9)	328 (14.4)	293 (18.0)	208 (25.4)	142 (31.4)
Skin or soft-tissue infection	919 (16.7)	5 (11.9)	54 (19.3)	395 (17.3)	272 (16.7)	140 (17.1)	53 (11.7)
Pneumonia or empyema^d^	694 (12.6)	1 (2.4)	15 (5.4)	203 (8.9)	206 (12.7)	150 (18.3)	119 (26.3)
Joint infection	531 (9.7)	2 (4.8)	29 (10.4)	239 (10.5)	169 (10.4)	66 (8.1)	26 (5.8)
Endocarditis	423 (7.7)	2 (4.85)	16 (5.7)	171 (7.5)	134 (8.2)	57 (7.0)	43 (9.5)
Peritonitis	140 (2.5)	1 (2.4)	9 (3.2)	68 (3.0)	41 (2.5)	16 (2.0)	5 (1.1)
Necrotizing fasciitis	114 (2.1)	0	14 (5.0)	65 (2.9)	26 (1.6)	8 (1.0)	1 (0.2)
Meningitis	78 (1.4)	0	5 (1.8)	34 (1.5)	23 (1.4)	7 (0.9)	9 (2.0)
Discordant	418 (7.6)	9 (21.4)	18 (6.4)	187 (8.2)	115 (7.1)	59 (0.6)	30 (6.6)

Abbreviation: BMI, body mass index (calculated as weight in kilograms divided by height in meters squared).

SI conversion factor: To convert hemoglobin A_1c_ to proportion of total hemoglobin, multiply by 0.01.

^a^ Includes American Indian, Alaska Native, Asian, Native Hawaiian or Pacific Islander, and unknown.

^b^ Includes unknown.

^c^ Some patients had multiple invasive infections, so the total number of infections exceeds the number of patients.

^d^ Includes 38 cases with group B *Streptococcus* recovered from pleural fluid.

Further assessment of patients with invasive GBS infections yielded insights into differences in underlying conditions and outcomes associated with each syndrome (Table 2). The most common comorbid conditions among patients with invasive GBS infections were diabetes (3364 [65.0%]), obesity (2669 [51.6%]), and chronic heart conditions (1633 [31.6%]). Those with osteomyelitis were most likely to experience recurrent infection (77 of 1075 [7.2%]) and had the highest prevalence of diabetes (928 [86.3%]) and peripheral vascular disease (342 [31.8%]). Among cases of osteomyelitis, 308 (28.7%) had *ICD* codes specifying involvement of the foot and/or ankle (data not shown). Patients with pneumonia or empyema were older, with a mean (SD) age exceeding 70 (11.8) years, were more likely to have 4 or more comorbid medical conditions (226 of 664 [34.0%]), and had higher prevalence of chronic heart conditions (271 [40.8%]), pulmonary conditions (267 [40.2%]), cancer (197 [29.7%]), and cerebrovascular disease (153 [23.0%]). Patients with peritonitis often had underlying chronic liver disease (64 of 138 [46.4%]).

**Table 2.** Characteristics and Outcomes of Patients in the Veterans Health Administration With Invasive Group B *Streptococcus* Infections by Clinical Syndrome

Characteristic	No. (%)										
	All (n = 5175)	Osteomyelitis (n = 1075)	Bacteremia Without Focus (n = 968)	Skin or Soft-Tissue Infection (n = 847)	Pneumonia or Empyema (n = 664)	Joint Infection (n = 501)	Endocarditis (n = 392)	Peritonitis (n = 138)	Necrotizing Fasciitis (n = 103)	Meningitis (n = 78)	Discordant Cases (n = 409)
Age, mean (SD), y	66.5 (11.7)	62.7 (10.3)	69.9 (12.5)	65.7 (11.0)	71.5 (11.8)	65.1 (10.3)	67.5 (11.6)	64.0 (10.1)	60.6 (9.6)	66.1 (11.4)	65.7 (11.8)
All-cause 30-d mortality	451 (8.7)	15 (1.4)	125 (12.9)	30 (3.5)	116 (17.5)	17 (3.4)	22 (5.6)	38 (27.5)	5 (4.9)	10 (12.8)	73 (17.8)
Recurrent infection	273 (5.3)	77 (7.2)	43 (4.4)	60 (7.1)	24 (3.6)	28 (5.6)	21 (5.4)	1 (0.7)	3 (2.9)	1 (1.3)	15 (3.7)
Polymicrobial infection	1400 (27.1)	628 (58.4)	176 (18.2)	110 (13.0)	124 (18.7)	76 (15.2)	54 (13.8)	31 (22.5)	41 (39.8)	10 (12.8)	150 (36.7)
* Staphylococcus aureus*	566 (10.9)	288 (26.8)	55 (5.7)	43 (5.1)	46 (6.9)	33 (6.6)	28 (7.1)	8 (5.8)	11 (10.7)	4 (5.1)	50 (12.2)
* Pseudomonas aeruginosa*	70 (1.4)	41 (3.8)	5 (0.5)	3 (0.4)	2 (0.3)	1 (0.2)	2 (0.5)	1 (0.7)	2 (1.9)	0	13 (3.2)
Comorbid conditions, No.											
0	386 (7.5)	22 (2.0)	83 (8.6)	72 (8.5)	43 (6.5)	68 (13.6)	23 (5.9)	10 (7.2)	11 (10.7)	15 (19.2)	39 (9.5)
1	1201 (23.2)	294 (27.3)	206 (21.3)	194 (22.9)	108 (16.3)	140 (27.9)	90 (23.0)	32 (23.2)	32 (31.1)	14 (17.9)	91 (22.2)
2-3	2324 (44.9)	512 (47.6)	423 (43.7)	400 (47.2)	287 (43.2)	202 (40.3)	171 (43.6)	64 (46.4)	41 (39.8)	30 (38.5)	194 (47.4)
≥4	1264 (24.4)	247 (23.0)	256 (26.4)	181 (21.4)	226 (34.0)	91 (18.2)	108 (27.6)	32 (23.2)	19 (18.4)	19 (24.4)	85 (20.8)
Specific comorbid conditions											
BMI ≥30	2669 (51.6)	528 (49.1)	440 (45.5)	563 (66.5)	320 (48.2)	303 (60.5)	187 (47.7)	68 (49.3)	54 (52.4)	35 (44.9)	171 (41.8)
Diabetes	3364 (65.0)	928 (86.3)	534 (55.2)	568 (67.1)	377 (56.8)	314 (62.7)	227 (57.9)	73 (52.9)	86 (83.5)	42 (53.8)	215 (52.6)
Chronic heart condition	1633 (31.6)	294 (27.3)	308 (31.8)	280 (33.1)	271 (40.8)	132 (26.3)	142 (36.2)	37 (26.8)	31 (30.1)	13 (16.7)	125 (30.6)
Renal disease	1427 (27.6)	300 (27.9)	284 (29.3)	229 (27.0)	210 (31.6)	103 (20.6)	125 (31.9)	35 (25.4)	25 (24.3)	16 (20.5)	100 (24.4)
Chronic pulmonary disease	1419 (27.4)	234 (21.8)	285 (29.4)	225 (26.6)	267 (40.2)	124 (24.8)	114 (29.1)	39 (28.3)	14 (13.6)	21 (26.9)	96 (23.5)
Peripheral vascular disease	1229 (23.7)	342 (31.8)	203 (21.0)	187 (22.1)	171 (25.8)	86 (17.2)	93 (23.7)	24 (17.4)	24 (23.3)	11 (14.1)	88 (21.5)
Cancer	1148 (22.2)	136 (12.7)	281 (29.0)	163 (19.2)	197 (29.7)	92 (18.4)	94 (24.0)	38 (27.5)	12 (11.7)	21 (26.9)	114 (27.9)
Cerebrovascular disease	832 (16.1)	141 (13.1)	181 (18.7)	115 (13.6)	153 (23.0)	55 (11.0)	87 (22.2)	11 (8.0)	8 (7.8)	15 (19.2)	66 (16.1)
Liver disease	772 (14.9)	105 (9.8)	158 (16.3)	107 (12.6)	101 (15.2)	67 (13.4)	73 (18.6)	64 (46.4)	6 (5.8)	18 (23.1)	73 (17.8)
Paralysis	335 (6.5)	118 (11)	74 (7.6)	30 (3.5)	40 (6.0)	20 (4.0)	14 (3.6)	4 (2.9)	2 (1.9)	7 (9.0)	26 (6.4)
Dementia	222 (4.3)	24 (2.2)	75 (7.7)	21 (2.5)	49 (7.4)	12 (2.4)	20 (5.1)	4 (2.9)	0	2 (2.6)	15 (3.7)
Peptic ulcer disease	203 (3.9)	26 (2.4)	40 (4.1)	30 (3.5)	36 (5.4)	15 (3.0)	20 (5.1)	13 (9.4)	1 (1.0)	5 (6.4)	17 (4.2)
Rheumatic disease	122 (2.4)	21 (2.0)	17 (1.8)	28 (3.3)	15 (2.3)	22 (4.4)	10 (2.6)	3 (2.2)	0	1 (1.3)	5 (1.2)
AIDS/HIV	69 (1.3)	8 (0.7)	14 (1.4)	12 (1.4)	10 (1.5)	6 (1.2)	5 (1.3)	3 (2.2)	0	3 (3.8)	8 (2.0)

Abbreviation: BMI, body mass index (calculated as weight in kilograms divided by height in meters squared).

Of 5175 cases of invasive GBS infection identified among VA users, 1400 (27.1%) were polymicrobial. *Staphylococcus aureus* and *P aeruginosa* were isolated in 566 (40.4%) and 70 (5.0%) polymicrobial cases, respectively. The proportion of polymicrobial cases varied by type of invasive GBS infection, ranging from 58.4% (628 of 1075) in cases of osteomyelitis to 15% or less in cases of meningitis (10 of 78 [12.8%]), endocarditis (54 of 392 [13.8%]), skin or soft-tissue infections (110 of 847 [13.0%]), and joint infection (76 of 501 [15.2%]).

### Mortality by Syndrome

**Figure 1.**
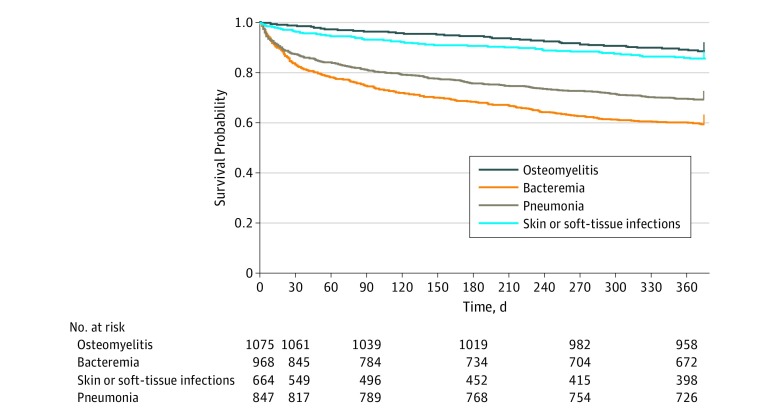
Kaplan-Meier Curves of Time to Death Following an Invasive Group B *Streptococcus* Infection by Infectious Syndrome

All-cause mortality at 30 days was 8.7% (451 of 5175) among patients with any type of invasive GBS infection; mortality was lowest among those with osteomyelitis (15 of 1075 [1.4%]) or joint infections (17 of 501 [3.4%]) and highest among patients with peritonitis (38 of 138 [27.5%]), followed by pneumonia or empyema (116 of 664 [17.5%]), bacteremia without focus (125 of 968 [12.9%]), and meningitis (10 of 78 [12.8%]). The overall 1-year mortality rate following any type of invasive GBS infection was 22.9% (1185 deaths among 5175 index cases). Survival curves of the of 4 most common invasive GBS infections showed decreased survival for those with pneumonia or empyema and bacteremia without focus, particularly in the first 90 days after infection (90 day mortality, 25.3% [168 of 664] and 19.1% [185 of 968], respectively). Mortality at 90 days was 3.3% (36 of 1075) among patients with osteomyelitis and 6.7% (58 of 847) among patients with skin and soft-tissue infections (Figure 1).

### Incidence Rates

**Figure 2.**
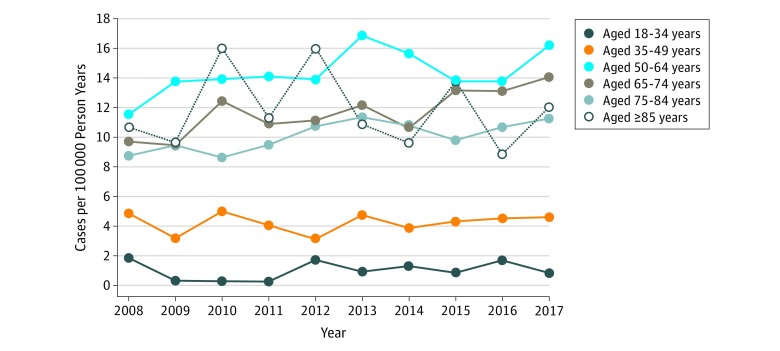
Incidence of Invasive Group B *Streptococcus* Infections Among Patients in the Veterans Health Administration, 2008-2017 Active Veterans Affairs health care users included those who had an outpatient primary care, specialty care clinic visit, or a hospital admission in the year evaluated. Invasive infections were defined as a positive culture from a normally sterile site.

Between 2008 and 2017, the incidence of invasive GBS infections among VA health care users increased from 9.23 cases per 100 000 person-years to 11.67 cases per 100 000 person-years (*P* = .049). The incidence of osteomyelitis increased from 1.2 cases per 100 000 person years in 2008 to 3.3 cases per 100 000 person-years in 2017 (*P* < .001). The incidence of infection did not appear to increase with age, with similar incidence rates observed among all age groups older than 50 years (Figure 2). However, after excluding VA health care users who were enrolled in Medicare, the incidence of invasive GBS infection increased according to age, in a pattern similar to that of estimates for the general US population^2^ (eFigure 1 in the Supplement).

**Figure 3.**
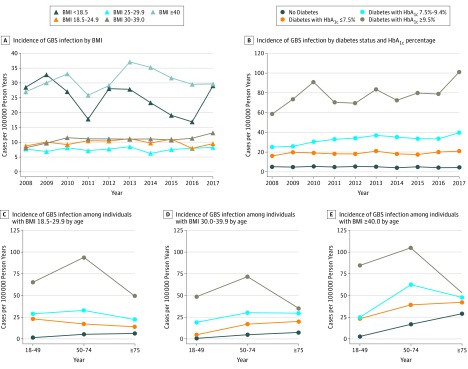
Incidence of Invasive Group B *Streptococcus* (GBS) Infections Among Patients in the Veterans Health Administration, Assessing Interactions Among Body Mass Index (BMI), Hemoglobin A_1c_ (HbA_1c_), and Age BMI calculated as weight in kilograms divided by height in meters squared. To convert HbA_1c_ to proportion of total hemoglobin, multiply by 0.01.

Stratification by BMI showed the highest incidence of invasive GBS infections among VA health care users at the extremes of BMI (BMI <18.5, 25.1 cases per 100 000 person-years; BMI ≥40, 31.0 cases per 100 000 person-years), while those with a BMI between 18.5 and 39.9 all had a similar, lower incidence (ie, 7.6-11.0 cases per 100 000 person-years) (Figure 3A; eTable 2 in the Supplement). Patients with BMI less than 18.5, who accounted for 2.1% (116 of 5497) of events, had a greater prevalence of chronic pulmonary conditions (47 of 113 [41.6%]) and paralysis (20 of 113 [17.7%]) and a lower prevalence of diabetes (38 of 113 [33.6%]) and heart conditions (26 of 113 [23.0%]) than patients with a BMI of 18.5 or greater (data not shown).

After aggregating cases and person-years across 10 years, the incidence of invasive GBS infections among VA health care users with diabetes and a HbA_1c_ level of 7.5% or lower was nearly 4-fold greater than that among patients without diabetes (19.0 [95% CI, 18.0-20.0] cases per 100 000 person-years vs 4.9 [95% CI, 4.7-5.2] cases per 100 000 person-years; incidence rate ratio, 3.9; 95% CI, 3.6-4.1; *P* < .001) (Figure 3B; eTable 2 in the Supplement). The incidence of invasive GBS infections increased among patients with diabetes with poor glycemic control. The incidence among those with an HbA_1c_ level of 7.5% to 9.4% was 33.2 (95% CI, 31.2-35.3) cases per 100 000 person-years, which increased to 78.3 (95% CI, 73.4-83.4) cases per 100 000 person-years among those with an HbA_1c_ level of 9.5% and higher (*P* < .001 for both rates compared with those with HbA_1c_ ≤7.5%). Compared with patients with well-controlled diabetes, those with an HbA_1c_ level of 9.5% or higher had an unadjusted incident rate ratio of 4.1 (95% CI, 3.4-4.4; *P* < .001).

Given that the rates of invasive GBS infections varied by age, BMI, and HbA_1c_ percentage, we calculated and plotted the rates across combinations of those variables to visualize their interactions (Figure 3C-E). Patients who were underweight (ie, BMI <18.5) were excluded. Among patients without diabetes, the rate of invasive GBS ranged from 0.9 (95% CI, 0.6-1.3) cases per 100 000 patient-years among patients aged 18 to 49 years with obesity to 29.0 (95% CI, 18.4-43.5) cases per 100 000 patient-years among patients aged 75 years or older with extreme obesity. Among patients with an HbA_1c_ level of at least 9.5%, the rates across age and BMI combinations ranged from 34.8 (95% CI, 20.6-54.9) cases per 100 000 person-years among patients aged 75 years or older with obesity to 104.9 (95% CI, 87.7-124.3) cases per 100 000 person-years among patients aged 50 to 74 years with extreme obesity. In every combination of age and BMI, the rate among patients with well-controlled diabetes was between the rate among patients with poorly controlled diabetes and patients without diabetes, ranging from 4.8 (95% CI, 2.1-9.4) cases per 100 000 person-years among patients aged 18 to 49 years with obesity to 42.2 (95% CI, 28.3-60.6) cases per 100 000 patient-years among patients aged 75 years or older with extreme obesity. Aggregating across all age and BMI groups, the rate among patients with well-controlled diabetes was significantly different from patients without diabetes and patients with poorly controlled diabetes (incidence rate ratio compared with patients without diabetes, 3.9; 95% CI, 3.6-4.1; *P* < .001; incidence rate ratio compared with patients with poorly controlled diabetes, 0.24; 95% CI, 0.22-0.26; *P* < .001). A similar pattern of increased incidence of invasive GBS infections among patients with higher HbA_1c_ percentage levels for the most common invasive GBS syndromes is detailed in eFigure 2 in the Supplement. A multivariable Poisson regression model estimated far higher incidence rate ratios for poor glycemic control (ie, HbA_1c_ ≥9.5%) than for advanced age or obesity (HbA_1c_ ≥9.5%: incidence rate ratio, 13.30; 95% CI, 12.26-14.41; aged ≥85 years: incidence rate ratio, 6.99; 95% CI, 5.10-9.87; BMI ≥40: incidence rate ratio, 2.37; 95% CI, 2.17-2.59) (eTable 2 in the Supplement).

## Discussion

In this large cohort of patients from the US VHA, the incidence of invasive GBS infection was associated with a diagnosis of diabetes and the degree of long-term glycemic control among those with diabetes. Patients with HbA_1c_ levels of 9.5% or higher had a 4-fold increase in the risk of invasive GBS compared with patients with HbA_1c_ levels less than 7.5%. Invasive GBS infection was also associated with BMI, with increased incidence occurring at the extremes of BMI (ie, <18.5 and ≥40). We uncovered differences in the underlying clinical characteristics and outcomes of different types of invasive GBS infection; patients who developed pneumonia or empyema were more likely to have cancer and chronic diseases of the heart, lungs, or kidneys and had a 30-day mortality of approximately 15%, comparable with that among patients with bacteremia without focus and meningitis. In contrast, patients with osteomyelitis (the most frequently observed infection in our cohort) were more likely to have diabetes and peripheral vascular disease, with a 30-day mortality rate of 1%, similar to that among patients with skin or soft-tissue infection.

In the United States and other countries, the most common syndromes of invasive GBS infections are bacteremia without focus and skin or soft-tissue infections.^2,7,8^ In VHA patients, the proportion of osteomyelitis among cases of invasive GBS infections was greater than in the general US population; furthermore, the incidence of osteomyelitis more than doubled between 2008 and 2017, from 1.2 to 3.3 cases per 100 000 person-years. Based on the analysis of *ICD* codes, at least 28% of those cases involved the foot and/or ankle and a high proportion (86%) of these patients had diabetes. Therefore, it is likely that many of these were diabetic foot infections with osteomyelitis, an important syndrome in the epidemiology of invasive GBS infections.^9^ In the VHA cohort, osteomyelitis and skin or soft-tissue infections were associated with recurrent infection, similar to a previous report of relapsing invasive GBS infections in adults.^10^

Invasive GBS infection is a burden for veterans, with some types of infection associated with substantial mortality. In VHA patients, the 30-day all-cause mortality rate among patients with invasive GBS infection was 8.7%. Although a different measure, this is comparable with the 6.5% case fatality of invasive GBS infection estimated by the ABCs program in the nonpregnant adult US population.^2^ Surprisingly, both necrotizing fasciitis and endocarditis involving GBS carried a lower risk of 30-day mortality compared with what is typically reported for these infections.^11,12,13^ In contrast, the greatest risk of mortality occurred among those with peritonitis and pneumonia or empyema. These syndromes were common in patients of advanced age and chronic diseases of the liver, heart, and lungs, contributing to their mortality. In discordant cases (where the diagnosis of infection was not captured in the electronic health record of patients with invasive GBS infection), there was a higher rate of mortality and cancer, suggesting that invasive GBS infection may have been only a single aspect of a complex and/or prolonged medical course or that patients were transferred to a non-VA hospital.

The increased risk of invasive GBS infection among patients with diabetes is well recognized in population-based surveillance studies in the United States.^1,2,3,4^ According to the ABCs program, diabetes is present in 53.4% of patients with invasive GBS infection, and diabetes confers the highest adjusted relative risk in young people without obesity.^4^ In the VHA cohort, where 66% of patients with invasive GBS infection had diabetes, the incidence of invasive GBS infection increased with rising HbA_1c_ percentages, a measure not available in population-based surveillance data. The increased incidence of invasive GBS infections among those with poorly controlled diabetes was consistent across age groups and patients who had normal weight, overweight, and obesity.

Data from ABCs also indicated that obesity is independently associated with an increased risk of invasive GBS infection. Specifically, Pitts et al^4^ reported a 5-fold higher risk in patients with a BMI of 40 or greater compared with patients with overweight. They also detected an interaction between diabetes and BMI in which the relative risk of infection for those with a BMI of 40 or greater was higher among patients without diabetes; patients with a BMI of less than 18.5 were excluded because of small numbers.^4^ Among VHA patients, there was a higher incidence of invasive GBS infection in patients with a BMI of 40 or greater as well as in patients with a BMI less than 18.5. Our multivariable modeling, which included long-term glycemic control among patients with diabetes, similarly indicated that extreme obesity was independently associated with an increased risk of invasive GBS infection, with an adjusted relative risk of 2.4 compared with individuals who had overweight.

Older adults with chronic medical conditions are known to be vulnerable to invasive GBS infection.^1,2,14,15,16,17,18^ Among VA health care users aged 18 to 64 years, rates of invasive GBS infection were similar to estimates for the US population; however, the rate of invasive GBS infections did not increase further in VA health care users aged 65 years or older. A potential explanation is that VA health care users aged 65 years or older who were enrolled in Medicare had access to health care services outside the VHA, and cases of invasive GBS infection in those patients were not captured by the VHA. When considering invasive GBS infections only among VA health care users who were not enrolled in Medicare, the rates of invasive GBS infections were similar to rates for the US population.^5^ Because VA health care users typically have a greater burden of chronic health conditions than their non-VA counterparts, it is unlikely that differences in baseline health status accounted for lower rates of invasive GBS infection in VA health care users aged 65 years or older.^19,20^ Indeed, there were comparable rates of diabetes, obesity, and other chronic medical conditions among VHA cases and the cases summarized in the ABCs reporting. While there is not yet a vaccine or protective measures specific to GBS infections for nonpregnant adults, VA health care may include preventative services, continuity of care, and earlier care of chronic illnesses that could affect GBS infection.^21,22^

### Limitations

This study has limitations. First, VA health care users are predominantly white and non-Latino men and have a different sociodemographic and health status than the rest of the US population,^19^ which limits the generalizability of these results. Specifically, the high burden of chronic medical conditions among VA health care users may overestimate the risk that these conditions pose for developing infections.^20^ However, findings from the VHA cohort are consistent with population-based studies that identified comorbid conditions as risk factors for invasive GBS infections.^1,2,3,4,14,23^ Second, individuals who were pregnant or post partum at the time of infection were not specifically excluded; this likely had negligible impact given that the study population was 97% men and most were aged 65 years or older. Third, we relied on definitions based on *ICD* codes as well as site of GBS culture to determine the type of invasive GBS infection. In particular, pneumonia, a common diagnosis among hospitalized patients, may be overrepresented. Our GBS culture data were obtained from microbiology tables within the VHA CDW, which are carefully curated. Additionally, our definitions, tailored to GBS infections, were similar to those previously described^24^ and did not impose categories for every GBS culture as indicated by the number of discordant or unclassified cases. Furthermore, we reviewed data from 200 patients (approximately 4% of cases) to verify the GBS culture data, infectious syndrome, and other details (eg, age, comorbid conditions, mortality). Fourth, individuals with an incident case occurring before the study period and those with a recurrent case occurring after the study period may affect the measured invasive GBS infection rate. The 10-year study period as well as the overall low rate of recurrent infection indicated these influences were minimal. Fifth, neither the serotypes nor antibiotic susceptibilities of GBS isolates were reported because these are not routinely determined by microbiology laboratories serving VA medical centers.

## Conclusions

This analysis of the epidemiology of invasive GBS infection among patients in the US VHA demonstrated that underlying characteristics and associated mortality vary among different syndromes and underscored that poor long-term glycemic control in patients with diabetes (determined by an elevated HbA_1c_ percentage) was an important and potentially modifiable risk factor. Efforts to reverse the increasing rates of invasive GBS infections in adults should continue to address diabetes, obesity, and other chronic medical conditions. Among those with diabetes, improving glycemic control may help mitigate the risk of invasive GBS infection.

## Notes

Supplement. **eFigure 1. **Comparison of Incidence of Invasive Group B *Streptococcal* Infections Among VHA Patients and Estimates in the US Population from the Active Bacterial Core Surveillance Program **eFigure 2. **Invasive Group B *Streptococcal* Infections Among VHA Patients, Stratified by Body Mass Index (BMI), Hemoglobin A_1c_ (HbA_1c_) Percentage, Age, and Infectious Syndrome **eTable 1. ***International Classification of Diseases* (*ICD*) Codes Used to Determine the Type of Invasive Group B *Streptococcal* Infection **eTable 2. **Crude Incidence Rates, Bivariate, and Multivariable Relative Risk of Invasive Group B *Streptococcus* Infection by Age, Body Mass Index, Diabetes Status, and Hemoglobin A_1c_ (HbA_1c_) Percentage
