# Supplementary material for: Risk Factors and Mortality Rates Associated With Invasive Group B Streptococcus Infections Among Patients in the US Veterans Health Administration
Source: JAMA Netw Open. 2019 Dec 27;2(12):e1918324. doi: 10.1001/jamanetworkopen.2019.18324 (PMC6991221; doi:10.1001/jamanetworkopen.2019.18324)
Supplement: Supplement. — eFigure 1. Comparison of Incidence of Invasive Group B Streptococcal Infections Among VHA Patients and Estimates in the US Population from the Active Bacterial Core Surveillance Program eFigure 2. Invasive Group B Streptococcal Infections Among VHA Patients, Stratified by Body Mass Index (BMI), Hemoglobin A1c (HbA1c) Percentage, Age, and Infectious Syndrome eTable 1. International Classification of Diseases (ICD) Codes Used to Determine the Type of Invasive Group B Streptococcal Infection eTable 2. Crude Incidence Rates, Bivariate, and Multivariable Relative Risk of Invasive Group B Streptococcus Infection by Age, Body Mass Index, Diabetes Status, and Hemoglobin A1c (HbA1c) Percentage [file jamanetwopen-2-e1918324-s001.pdf]

Supplementary Online Content

Jump RLP, Wilson BM, Baechle D, et al. Risk factors and mortality rates associated with invasive group B *Streptococcus* infections among patients in the US Veterans Health Administration. *JAMA Netw Open*. 2019;2(12):e1918324. doi:10.1001/jamanetworkopen.2019.18324

eFigure 1. Comparison of Incidence of Invasive Group B *Streptococcal* Infections Among VHA Patients and Estimates in the US Population from the Active Bacterial Core Surveillance Program
eFigure 2. Invasive Group B *Streptococcal* Infections Among VHA Patients, Stratified by Body Mass Index (BMI), Hemoglobin A_{1c} (HbA_{1c}) Percentage, Age, and Infectious Syndrome
eTable 1. *International Classification of Diseases* (*ICD*) Codes Used to Determine the Type of Invasive Group B *Streptococcal* Infection
eTable 2. Crude Incidence Rates, Bivariate, and Multivariable Relative Risk of Invasive Group B *Streptococcus* Infection by Age, Body Mass Index, Diabetes Status, and Hemoglobin A_{1c} (HbA_{1c}) Percentage

This supplementary material has been provided by the authors to give readers additional information about their work.

© 2019 Jump RLP et al. *JAMA Network Open.*

eFigure 1. Comparison of Incidence of Invasive Group B *Streptococcal* Infections Among VHA Patients and Estimates in the US Population from the Active Bacterial Core Surveillance Program

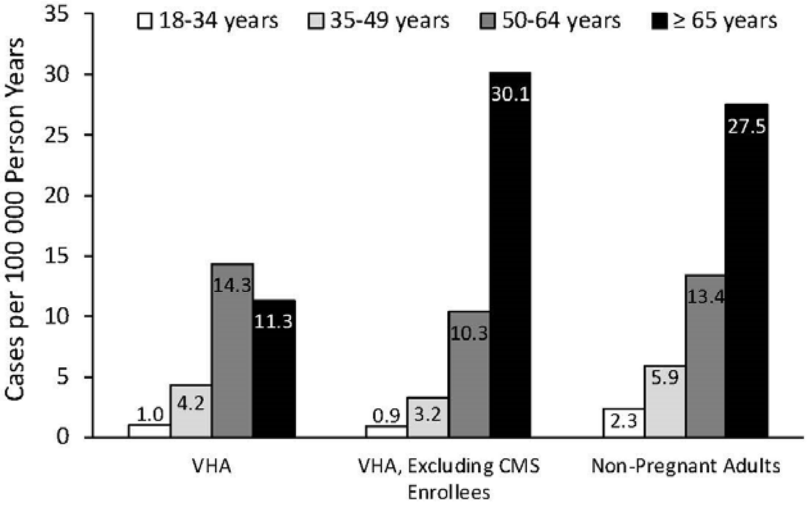

The first set of bars (VHA) includes all veterans who were active in the Veterans Health Administration (VHA) for the year assessed. The second set of bars (VHA, Excluding CMS Enrollees) includes active veterans who were not enrolled in Centers for Medicare or Medicaid Services (CMS) for the year assessed. The third set of bars (Non-Pregnant Adults) shows the rates for non-pregnant adults as reported by the US Center for Disease Control and Prevention's Active Bacterial Core surveillance (website accessed 5/16/19).

© 2019 Jump RLP et al. *JAMA Network Open.*

eFigure 2. Invasive Group B *Streptococcal* Infections Among VHA Patients, Stratified by Body Mass Index (BMI), Hemoglobin A_{1c} (HbA_{1c}) Percentage, Age, and Infectious Syndrome

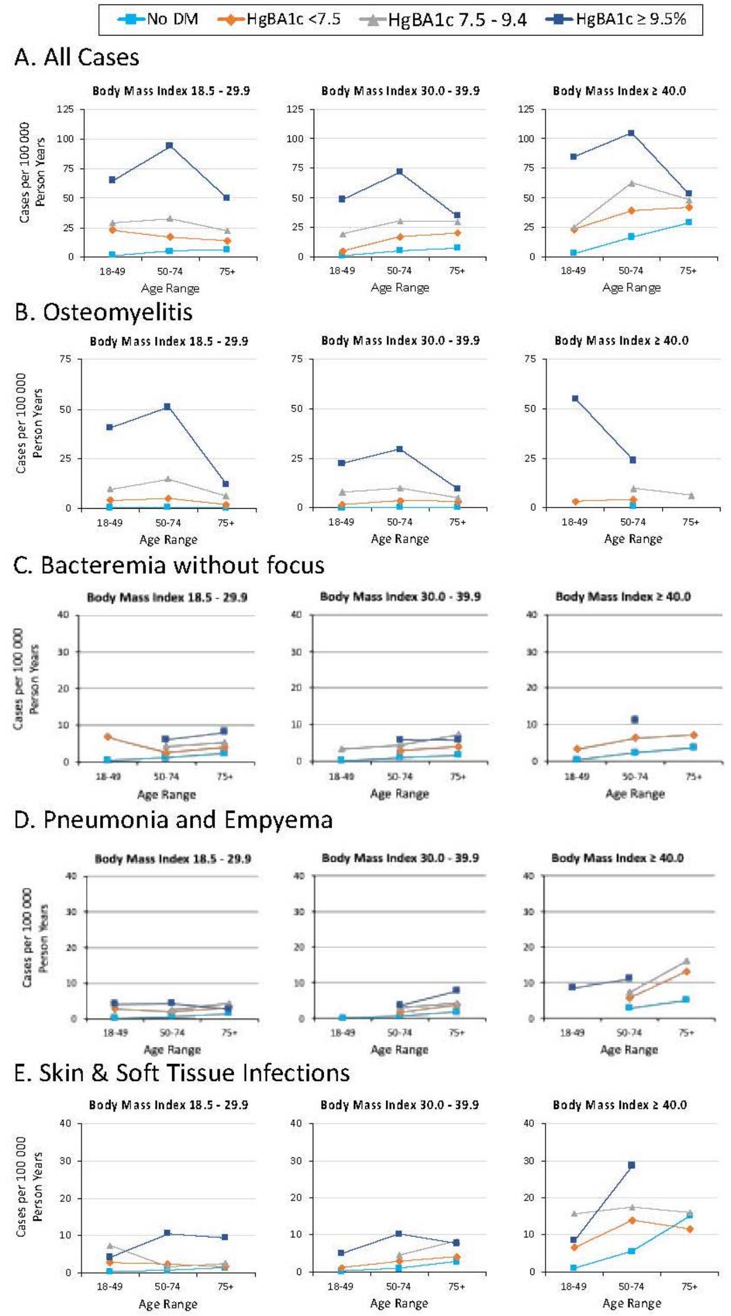

Incomplete and missing lines are due to an insufficient number of cases or patient years at risk (< 2 cases or < 1,000 person-years at risk) for some of the categories of age or body mass index to calculate a rate for specific types of invasive GBS infections.

© 2019 Jump RLP et al. *JAMA Network Open.*

eTable 1. *International Classification of Diseases* (*ICD*) Codes Used to Determine the Type of Invasive Group B *Streptococcal* Infection.

Syndrome	ICD9 Code	ICD10 Code
Bacteremia	038.0, 038.8, 038.9, 415.12, 449, 771.81, 771.83, 790.7, 999.32	I26.01, I26.90, I76., O88.311, O88.312, O88.313, O88.319, O88.32, O88.33, R78.81, T80.211A, T80.211D, T80.212A, T80.211S, T86.03,
Endocarditis	422.92, 424.90, 424.91, 424.99, 429.0, 996.61, 996.71, 420.0, 420.90, 420.91, 420.99, 421.0, 421.1, 421.9	I30.1, I30.9, I33.0, I33.9, I40.1, I40.8, I38., I39., I40.0, I40.9, I41., I51.4, T82.6XXA, T82.6XXD, T82.6XXS, T86.23, T86.33
Joint infection	726.33, 726.61, 726.62, 726.63, 726.65, 726.71, 727.3, 711.00, 711.01, 711.02, 711.03, 711.04, 711.05, 711.06, 711.07, 711.08, 711.09, 711.40, 711.41, 711.42, 711.43, 711.44, 711.45, 711.46, 711.47, 711.48, 711.49, 711.81, 711.86, 711.90, 711.91, 711.92, 711.93, 711.94, 711.95, 711.96, 711.97, 711.98, 711.99, 996.66, 996.67, 711.80, 711.82, 711.83, 711.84, 711.85, 711.87, 711.88, 711.89, 719.00, 719.01, 719.02, 719.03, 719.04, 719.05, 719.06, 719.07, 719.08, 719.09	M65.121, M65.122, M65.129, M65.131, M65.132, M65.139, M65.079, M65.142, M65.149, M65.151, M65.152, M65.159, M65.08, M65.141, M65.169, M65.161, M65.162, M65.179, M65.18, M65.19, M65.171, M65.172, M70.21, M70.22, M70.41, M70.42, M70.52, M70.61, M70.62, M70.71, M70.72, M71.00, M71.011, M71.012, M71.019, M71.021, M71.022, M71.029, M71.031, M71.032, M71.039, M71.041, M71.042, M71.049, M71.051, M71.052, M71.059, M71.061, M71.062, M71.069, M71.071, M71.072, M71.079, M71.10, M71.111, M71.112, M71.119, M71.08, M71.09, M71.129, M71.131, M71.132, M71.139, M71.141, M71.142, M71.149, M71.121, M71.152, M71.159, M71.122, M71.151, M71.169, M71.171, M71.172, M71.179, M71.18, M71.19, M71.161, M71.162, M70.30, M70.31, M70.32, M70.40, M70.50, M70.51, M70.60, M70.70, M65.10, M65.111, M65.112, M65.119, M00.20, M00.211, M00.212, M00.219, M00.221, M00.222, M00.229, M00.231, M00.232, M00.239, M00.241, M00.242, M00.249, M00.251, M00.252, M00.259, M00.261, M00.262, M00.269, M00.271, M00.272, M00.279, M00.28, M00.29, M00.80, M00.811, M00.812, M00.819, M00.821, M00.822, M00.829, M00.831, M00.832, M00.839, M00.841, M00.842, M00.849, M00.851, M00.852, M00.859, M00.861, M00.862, M00.869, M00.871, M00.872, M00.879, M00.88, M00.89, M01.X11, M01.X12, M01.X19, M01.X21, M01.X22, M01.X29, M01.X31, M01.X32, M01.X39, M00.9, M01.X42, M01.X49, M01.X51, M01.X52, M01.X59, M01.X61, M01.X62, M01.X69, M01.X0, M01.X41, M01.X71, M01.X8, M01.X9, M01.X72, M01.X79

© 2019 Jump RLP et al. *JAMA Network Open.*

Syndrome	ICD9 Code	ICD10 Code
Meningitis	996.63, 325., 326., 320.2, 320.7, 320.8, 320.89, 320.9, 322.9, 324.0, 324.1, 324.9	G00.2, G00.8, G00.9, G01., G03.1, G03.8, G03.9, G04.2, G06.0, G06.1, G06.2, G07., T85.730A, T85.730D, T85.730S, T85.735A, T85.735D, T85.735S
Necrotizing fasciitis	729.4, 728.86, 040.0	M72.6, N49.3
Osteomyelitis	376.03, 730.00, 730.01, 730.02, 730.03, 730.04, 730.05, 730.06, 730.08, 730.09, 730.10, 730.11, 730.12, 730.13, 730.14, 730.15, 730.16, 730.18, 730.19, 730.20, 730.21, 730.22, 730.23, 730.24, 730.25, 730.26, 730.28, 730.29, 730.80, 730.81, 730.82, 730.83, 730.84, 730.85, 730.86, 730.88, 730.89, 730.90, 730.91, 730.92, 730.93, 730.94, 730.95, 730.96, 730.98, 730.99	H05.021, H05.022, H05.023, H05.029, M46.22, M46.23, M46.24, M46.25, M46.26, M46.30, M46.31, M46.32, M46.33, M46.34, M46.35, M46.27, M46.37, M46.38, M46.39, M46.28, M46.36, M46.50, M46.51, M46.52, M46.53, M46.54, M46.55, M46.56, M46.57, M46.58, M46.59, M86.00, M86.011, M86.012, M86.019, M86.021, M86.022, M86.029, M86.031, M86.032, M86.039, M86.041, M86.042, M86.049, M86.051, M86.052, M86.059, M86.061, M86.062, M86.069, M86.08, M86.09, M86.10, M86.111, M86.112, M86.119, M86.121, M86.122, M86.129, M86.131, M86.132, M86.139, M86.141, M86.142, M86.149, M86.151, M86.152, M86.159, M86.161, M86.162, M86.169, M86.18, M86.19, M86.20, M86.211, M86.212, M86.219, M86.221, M86.222, M86.229, M86.231, M86.232, M86.239, M86.241, M86.242, M86.249, M86.251, M86.252, M46.80, M46.81, M86.259, M86.261, M86.262, M86.269, M86.28, M86.29, M86.30, M86.311, M86.312, M86.319, M86.321, M86.322, M86.329, M86.331, M86.332, M86.339, M86.341, M86.342, M86.349, M86.351, M86.352, M86.359, M86.361, M86.362, M86.369, M86.38, M86.39, M86.40, M86.411, M86.412, M86.419, M86.421, M86.422, M86.429, M86.431, M86.432, M86.439, M86.441, M86.442, M86.449, M86.451, M86.452, M86.459, M86.461, M86.462, M86.469, M86.48, M86.49, M86.50, M86.511, M86.512, M86.519, M86.521, M86.522, M86.529, M86.531, M86.532, M86.539, M86.541, M86.542, M86.549, M86.551, M86.552, M86.559, M86.561, M86.562, M86.569, M86.58, M86.59, M86.60, M86.611, M86.612, M86.619, M86.621, M86.622, M86.629, M86.631, M86.632, M86.639, M86.641, M86.642, M86.649, M86.651, M86.652, M86.659, M86.661, M86.662, M86.669, M86.68, M86.69, M86.8X0, M86.8X1, M86.8X2, M86.8X3, M86.8X4, M86.8X5, M86.8X6, M86.8X8, M86.8X9, M86.9, T86.832, M46.20, M46.21, M46.40, M46.41, M46.43, M46.45, M46.42, M46.47, M46.44, M46.49, M46.46, M46.48
Osteomyelitis, foot/ankle	730.07, 730.17, 730.27, 730.87, 730.97	M86.671, M86.672, M86.679, M86.8X7, M86.071, M86.072, M86.079, M86.171, M86.172, M86.179,

Syndrome	ICD9 Code	ICD10 Code
		M86.271, M86.272, M86.279, M86.371, M86.372, M86.379, M86.471, M86.472, M86.479, M86.571, M86.572, M86.579
Peritonitis	567.22, 567.31, 601.2, 289.2, 530.86, 536.41, 539.81, 540.0, 540.1, 562.01, 562.11, 562.13, 574.00, 574.01, 574.10, 574.11, 574.30, 574.31, 574.40, 574.41, 574.51, 574.60, 574.61, 574.70, 574.71, 574.80, 574.81, 574.90, 574.91, 575.0, 575.1, 575.10, 575.12, 576.1, 567.0, 567.2, 567.21, 567.23, 567.29, 567.38, 567.39, 567.8, 567.81, 567.89, 567.9, 569.5, 569.71, 590.2, 572.1	D73.3, I88.0, K35.2, K35.3, K35.80, K50.014, K50.114, K50.814, K50.914, K51.014, K51.214, K51.314, K51.414, K51.514, K51.814, K51.914, K36., K37., K55.30, K55.31, K55.32, K55.33, K56.5, K57.00, K57.01, K57.12, K57.13, K57.20, K57.21, K57.31, K57.32, K57.33, K57.40, K57.41, K57.52, K57.53, K57.80, K57.81, K57.92, K61.0, K61.1, K61.2, K63.0, K61.3, K61.4, K65.0, K65.1, K65.2, K65.3, K65.4, K65.9, K66.0, K68.11, K68.12, K68.19, K75.0, K75.1, K81.0, K81.1, K81.2, K81.9, K83.0, K85.02, K85.12, K95.01, K85.82, K91.850, P77.2, P77.3, P77.9, T85.71XA, T85.71XD, T85.71XS, T86.852, T86.43
Pneumonia/ empyema	510.0, 510.9, 511.1, 513.1, 513.0, 482.32, 482.3, 482.30, 482.39, 482.8, 482.89, 482.9, 485., 486., 483., 483.8, 484.8, 487.0, 516.30, 997.31, 997.32	J86.0, J86.9, J85.3, J90., J15.3, P23.3, J15.4, J16.8, J15.8, J15.9, J17., J18.0, J18.1, J18.8, J18.9, J22., J44.0, J85.0, J85.1, J85.2, J95.851, P23.6, T86.812
Sepsis, Toxic Shock	995.91, 995.91, 995.92, 995.92, 998.02, 040.82, 785.52	A40.1, P36.0, A40.8, A40.9, A41.89, A41.9, O85., P36.10, P36.19, P77.1, P36.8, P36.9, R57.9, R65.10, R65.20, R65.21, T81.12XA, T81.12XD, T81.12XS, A48.3
Skin/soft tissue infection	616.4, 566., 675.10, 675.11, 675.12, 675.13, 675.14, 681.9, 682.0, 682.1, 682.2, 682.3, 682.4, 682.5, 682.6, 682.7, 682.8, 682.9, 685.0, 728.0, 572.0, 569.61, 729.30, 729.39, 785.4, 035., 675.00, 675.01, 675.02, 675.03, 675.04, 675.20, 675.21, 675.22, 675.23, 675.24, 675.80, 675.81, 675.82, 675.83, 675.84, 675.90, 675.91, 675.92, 675.93,	N99.511, N99.521, N99.521, N99.531, N99.531, A46., A48.0, E08.52, E09.52, E10.52, E11.52, E13.52, H00.031, H00.032, H00.033, H00.034, H00.035, H00.036, H00.039, H05.011, H05.012, H05.013, H05.019, H60.00, H60.01, H60.02, H60.03, H60.10, H60.11, H60.12, H60.13, I88.1, I88.8, I88.9, I96., J34.0, L01.00, L01.01, L01.03, L01.09, L02.01, L02.02, L02.03, L02.11, L02.12, L02.13, L02.211, L02.212, L02.213, L02.214, L02.215, L02.221, L02.216, L02.219, L02.222, L02.223, L02.226, L02.224, L02.231, L02.225, L02.229, L02.232, L02.235, L02.236, L02.233, L02.234, L02.239, L02.31, L02.32, L02.33, L02.411, L02.412, L02.413, L02.414, L02.415, L02.416, L02.419, L02.421, L02.422, L02.423, L02.424, L02.425, L02.426, L02.429, L02.431, L02.432, L02.433, L02.434, L02.435, L02.436, L02.439, L02.511, L02.521, L02.522, L02.529,

© 2019 Jump RLP et al. *JAMA Network Open.*

Syndrome	ICD9 Code	ICD10 Code
	675.94, 680.0, 680.1, 680.2, 680.3, 680.4, 680.5, 680.6, 680.7, 680.8, 680.9, 681.00, 681.01, 681.02, 681.10, 681.11, 683., 684., 685.1, 686.0, 686.00, 686.01, 686.09, 686.1, 686.8, 686.9, 705.83, 958.3, 997.62, 051.2, 289.1, 607.1, 911.9, 912.1, 912.9, 913.1, 913.3, 913.5, 913.7, 913.9, 914.1, 914.3, 914.5, 914.7, 914.9, 915.1, 915.5, 915.7, 915.9, 916.1, 916.5, 916.7, 916.9, 917.1, 917.3, 917.5, 917.7, 917.9, 919.1, 919.3, 919.5, 919.7, 919.9, 596.81, 998.5, 998.51, 998.59, 597.0	L02.531, L02.532, L02.539, L02.512, L02.519, L02.611, L02.612, L02.619, L02.621, L02.622, L02.629, L02.631, L02.632, L02.639, L02.811, L02.828, L02.818, L02.821, L02.831, L02.838, L02.91, L02.92, L02.93, L03.011, L03.012, L03.019, L03.031, L03.032, L03.039, L03.111, L03.112, L03.113, L03.114, L03.115, L03.116, L03.119, L03.211, L03.213, L03.221, L03.311, L03.312, L03.313, L03.314, L03.315, L03.316, L03.317, L03.319, L03.811, L03.818, L03.90, L04.0, L04.1, L04.2, L04.3, L04.8, L04.9, L05.01, L05.02, L08.89, L08.9, L30.3, M60.001, M60.002, N48.22, N48.5, N61.0, N61.1, O86.0, O91.011, O91.012, O91.013, O91.019, O91.02, O91.03, O91.111, O91.112, O91.113, O91.119, O91.12, O91.13, T87.41, T86.822, T87.40, T87.42, T87.43, T87.44, M60.000, M60.005, M60.009, M60.011, M60.012, M60.019, M60.003, M60.004, M60.029, M60.031, M60.032, M60.039, M60.041, M60.042, M60.043, M60.044, M60.045, M60.046, M60.021, M60.052, M60.059, M60.022, M60.062, M60.069, M60.070, M60.071, M60.072, M60.073, M60.051, M60.075, M60.061, M60.074, M60.078, M60.076, M60.077, M60.08, M60.09, M60.9, M65.00, M65.011, M65.012, M65.019, M65.021, M65.022, M65.029, M65.031, M65.032, M65.039, M65.041, M65.042, M65.049, M65.051, M65.052, M65.059, M65.061, M65.062, M65.069, M65.071, M65.072

© 2019 Jump RLP et al. *JAMA Network Open.*

eTable 2. Crude Incidence Rates, Bivariate, and Multivariable Relative Risk of Invasive Group B *Streptococcus* Infection by Age, Body Mass Index, Diabetes Status, and Hemoglobin A_{1c} (HbA_{1c}) Percentage

**Variable**	**Crude Incidence Rate (95% confidence interval (CI))**	**Univariate Poisson Model Risk Ratio (95% CI)**	**Multivariable Poisson Model Risk Ratio (95%CI)**
Age (years)			
18-34	1.03 (0.74,1.4)	Reference	Reference
35-49	4.25 (3.76,4.79)	4.13 (3.01,5.81)	2.75 (1.99, 3.92)
50-64	14.29 (13.71,14.89)	13.88 (10.34,19.21)	6.48 (4.79, 9.06)
65-74	11.95 (11.37,12.54)	11.61 (8.64,16.08)	5.30 (3.91, 7.42)
75-84	10.03 (9.36,10.74)	9.74 (7.22,13.54)	5.18 (3.81, 7.28)
85+	11.91 (10.84,13.06)	11.57 (8.52,16.17)	6.99 (5.10, 9.87)
Body Mass Index[a]			
Normal (18.5-24.9)	9.85 (9.24, 10.49)	1.29 (1.19, 1.40)	1.51 (1.39, 1.64)
Underweight (< 18.5)	25.05 (20.70, 30.05)	3.28 (2.70, 3.95)	3.97 (3.26, 4.77)
Overweight (25-29.9)	7.64 (7.24, 8.04)	Reference	Reference
Obese (30-39.9)	11.01 (10.54, 11.50)	1.44 (1.35, 1.54)	1.12 (1.05, 1.20)
Extremely obese (≥ 40)	30.97 (28.92, 33.13)	4.06 (3.73, 4.42)	2.37 (2.17, 2.59)
Diabetes Mellitus (DM) Status and Hemoglobin A1c (HbA1c)[a]			
No DM	4.92 (4.70, 5.15)	Reference	Reference
DM, HbA1c < 7.5%	18.98 (18.00, 20.00)	3.86 (3.60, 4.13)	3.26 (3.04, 3.51)
DM, HbA1c 7.5-9.4%	33.17 (31.18, 35.25)	6.74 (6.25, 7.27)	5.64 (5.21, 6.11)
DM, HbA1c ≥ 9.5%	78.28 (73.42, 83.38)	15.91 (14.72, 17.19)	13.30 (12.26, 14.41)

[a]Individuals without a BMI or HbA1c were excluded.

© 2019 Jump RLP et al. *JAMA Network Open.*
